# Machine learning analysis of student consumer choices for pet supplements

**DOI:** 10.3389/frai.2026.1764233

**Published:** 2026-06-03

**Authors:** Ta-Chen Chen, Hui Yu Chung, Fu Shih Chen

**Affiliations:** 1 Graduate School of Pharmaceutical Sciences, Nihon Pharmaceutical University, Tokyo, Japan; 2 Center for General Education and Master Program of Digital Health Innovation, College of Humanities and Sciences, China Medical University, Taichung, Taiwan; 3 Faculty of Pharmaceutical Sciences, Nihon Pharmaceutical University, Saitama, Japan

**Keywords:** Complementary and Alternative Medicine, consumer behavior, decision tree, machine learning, pet owner perception, pet supplements

## Abstract

**Background:**

Motivated by the rising global trend of veterinary Complementary and Alternative Medicine (CAM) usage and a specific data gap in Taiwan, this study investigates the consumption behavior of future pet owners.

**Methods:**

A cross-sectional survey was conducted among Taiwanese medical university students using a validated online questionnaire. Beyond traditional descriptive statistics, this study employed machine learning techniques to analyze owner demographics, pet characteristics, and determinants of CAM usage.

**Results:**

Data analysis revealed a strong correlation between positive owner perceptions—specifically satisfaction, belief in benefits, and understanding—and targeted CAM application. A decision tree model successfully identified “overall satisfaction” as the primary splitting criterion for user segmentation, followed by belief and understanding. Predictive modeling demonstrated high accuracy in identifying usage motivations for joint and digestive health, though predicting “immune system boosting” proved more complex due to behavioral variability.

**Conclusion:**

Owner satisfaction is the critical predictor of CAM usage patterns. While the predictive model for specific conditions, such as joint and digestive health, yielded high accuracy (AUC > 0.93), these findings should be interpreted as an exploratory framework given the pilot nature of the study and the limited sample size (*n* = 41). These findings suggest that veterinarians and industry stakeholders should adopt data-driven communication strategies focusing on transparency and satisfaction.

## 1 Introduction

With the growing human-animal bond, more pets are becoming part of families, thereby fostering the concept of living together, and alternative complementary therapies for pets have become more popular (Fine et al., 2019). Thus, Complementary and Alternative Medicine (CAM) and supplements are increasingly integrated into veterinary clinical practices through homeopathy, phytotherapy, and biophysical interventions (Stanossek and Wehrend, 2022). Pet CAM encompasses a diverse range of diagnostic, therapeutic, and preventive practices, which have led to the transformation of conventional veterinary medicine into its present popularity (Gilberg et al., 2021). There is a growing global trend among pet owners to express interest in and use Complementary and Alternative Medicine (CAM) for animals, often referred to as Complementary and Alternative Veterinary Medicine (CAVM) (Gilberg et al., 2021). This trend is evident not only in European countries like Sweden (Gilberg et al., 2021), Germany (Stanossek and Wehrend, 2022; Stanossek and Wehrend, 2023), and Spain (Romero et al., 2022), but also in other regions worldwide. Many pet owners seek CAM as a complementary or alternative approach to conventional veterinary treatments (Miscioscia and Repac, 2022). This reflects a rising interest in holistic health and natural therapies for their animal companions.

PubMed data found a general increase in research on non-conventional therapies (NCTs) over the past 20 years, particularly on plant extracts and essential oils (Domingues et al., 2022). A study finds that 80% of Spanish small animal veterinarians use medicinal plants, such as cannabis, aloe, and thyme, especially for musculoskeletal and gastrointestinal disorders (Romero et al., 2022). In Germany, a survey of small animal veterinarians showed that 85.4% use natural and complementary therapies, the most common being complex homeopathy, phytotherapy, classical homeopathy, and biophysical therapy. The most frequently treated conditions include orthopedic, geriatric, and metabolic diseases. Additionally, 57.9% of veterinarians reported an increasing demand for natural and complementary therapies from pet owners over the past 5 years. German veterinarians primarily obtain CAM information through scientific journals and continuing education courses (Stanossek and Wehrend, 2022).

The use of CAVM is widespread in Europe, with phytotherapy, homeopathy, acupuncture, and other therapies being quite common (Stanossek and Wehrend, 2022; Stanossek and Wehrend, 2023). Veterinary schools in the US are increasingly incorporating CAM education and research into their curricula (Schoen, 2000; Memon and Sprunger, 2011). Recent studies indicate that nutritional therapy, phytotherapy, acupuncture, and physical therapy are the most commonly integrated CAM topics into veterinary curricula (Memon and Sprunger, 2011). Veterinary schools in Canada are also beginning to offer CAVM coursework (Memon and Sprunger, 2011). This suggests a growing recognition of the potential benefits of these therapies in veterinary practice. Veterinary schools in Australia are also beginning to offer CAM courses (Memon and Sprunger, 2011).

Traditional Japanese medicine (Kampo) has been found to have applications in veterinary practice in Asia. Herbal formulations like Daikenchuto and Juzen-taiho-to are used to improve gastrointestinal motility (Shinohara et al., 2022; Shinohara et al., 2019a; Yanai et al., 2013; Maeda et al., 2020). Studies have shown that Juzen-taiho-to can promote gastrointestinal motility in dogs and may have antioxidant effects (Shinohara et al., 2019a; Shinohara et al., 2019b). As previously mentioned, there is a lack of data on CAM usage, especially in phytotherapy in Taiwan. Currently, we cannot ascertain the actual prevalence of herbal supplements used among Taiwanese, not only among pet owners but also among potential future pet owners among university students, nor the popularity of specific therapies. Thus, further research is needed to understand Taiwan’s current landscape of Complementary and Alternative Medicine health supplements.

Therefore, this study aims to investigate the key predictive factors influencing pet owners’ CAM supplement choices, based on their cognitive attitudes. Specifically, we hypothesize that pet owners’ overall satisfaction, perceived benefits, and understanding of the products are significant predictors of their reasons for using CAM supplements. The questionnaire was used in a research project to gather information on pet owners’ usage and opinions on Complementary and Alternative Medicine (CAM) health supplements for their pets. It asks questions about pet owners’ demographics, ownership history, CAM supplement awareness, usage patterns, and satisfaction with CAM products.

## 2 Materials and methods

### 2.1 Study design and participants

This cross-sectional study was conducted among students at a medical university in Taiwan. Participants were recruited through Google Forms and social media groups across different majors at the university. The inclusion criteria were: (1) being a currently enrolled student at the medical university, and (2) being 18 years of age or older. The characteristics of the 328 participants are detailed in Table 1. The sample predominantly consisted of females (58.53%), with a majority aged between 18 and 25 (41.16%). The most frequent duration of ownership was less than 1 year in the age group between 18 and 25 (39.6.0%).

**Table 1:** The basic characteristics of campus participants with questionnaire.

Variable	Age						
	≦18ys	18–25ys	25–35ys	35–45ys	45–55ys	>55ys~	*p*-value^*^
Gender (*n*, %)							0.4088
Female	19 (5.79)	135 (41.16)	33 (10.06)	4 (1.22)	1 (0.3)	0 (0)	
Male	11 (3.35)	74 (22.56)	44 (13.41)	2 (0.61)	2 (0.61)	1 (0.3)	
Other	0 (0)	2 (0.61)	0 (0)	0 (0)	0 (0)	0 (0)	
Education level (*n*, %)							
Bachelor’s degree	3 (0.91)	53 (16.16)	67 (20.43)	3 (0.91)	0 (0)	0 (0)	<0.0001
Master’s degree	0 (0)	1 (0.3)	10 (3.05)	3 (0.91)	2 (0.61)	0 (0)	
High school or below	27 (8.23)	157 (47.87)	0 (0)	0 (0)	1 (0.3)	1 (0.3)	
Occupation (*n*, %)							<0.0001
Office worker/White-collar	0 (0)	1 (0.3)	6 (1.83)	3 (0.91)	2 (0.61)	0 (0)	
Self-employed/Entrepreneur	0 (0)	0 (0)	0 (0)	0 (0)	1 (0.30)	0 (0)	
Student	30 (9.15)	210 (64.02)	71 (21.65)	3 (0.91)	0 (0)	1 (0.30)	
Annual income (*n*, %)							<0.0001
Under NT$400,000	29 (8.84)	206 (62.80)	67 (20.43)	2 (0.61)	1 (0.30)	1 (0.30)	
NT$410,000–NT$700,000	1 (0.30)	1 (0.30)	7 (2.13)	0 (0)	1 (0.30)	0 (0)	
NT$710,000–NT$1,000,000	0 (0)	3 (0.91)	3 (0.91)	1 (0.30)	0 (0)	0 (0)	
NT$1,010,000–NT$1,300,000	0 (0)	1 (0.30)	0 (0)	1 (0.30)	0 (0)	0 (0)	
Above NT$1,310,000	0 (0)	0 (0)	0 (0)	2 (0.61)	1 (0.30)	0 (0)	
Your total years of pet ownership experience (*n*, %)							0.0134
Less than 1 year	12 (4.80)	99 (39.60)	30 (12.00)	1 (0.40)	0 (0)	1 (0.40)	
1–3 years	4 (1.60)	27 (10.80)	7 (2.80)	2 (0.80)	0 (0)	0 (0)	
4–6 years	0 (0)	16 (6.40)	10 (4.00)	1 (0.40)	0 (0)	0 (0)	
7–10 years	2 (0.80)	10 (4.00)	3 (1.20)	1 (0.40)	1 (0.40)	0 (0)	
Over 10 years	1 (0.40)	7 (2.80)	12 (4.80)	1 (0.40)	2 (0.80)	0 (0)	

* The *p*-value was estimated from the likelihood test using the contingency analysis.

### 2.2 Data collection

Data were collected using a self-administered online questionnaire adapted from a previously validated survey on the perceptions and usage of Complementary and Alternative Medicine (CAM) health supplements for pets among both pet owners and non-pet owners (see Supplementary Table 1). The questionnaire consisted of 50 items divided into seven sections:

- Survey Description: Briefly explained the purpose of the study and assured respondents of their anonymity.
- Basic Information of Owner: Collected demographic data, including age, gender, education level, place of residence, occupation, and annual income.
- Pet Information of Owner: Gathered information on pet ownership, including years of experience, number and types of pets owned, breeds, and the current health status of pets.
- Awareness and Acceptance of CAM Health Supplements for Pets: Assessed participants’ familiarity with CAM supplements, sources of information, perceived benefits, understanding of ingredients and effects, and willingness to use CAM for their pets.
- Actual Usage of CAM Health Supplements for Pets: Analyzed past and present usage patterns of CAM health supplements, including product names, duration of use, frequency, purchase locations, reasons for use, and monthly expenditures on CAM supplements.
- Evaluation of CAM Health Supplement Effects: Explored pet owners’ perceptions of the effectiveness of CAM supplements in improving various aspects of their pets’ health, including immunity, digestion, skin and coat condition, and stress levels. This section also inquired about preferred dosage forms, overall satisfaction, perceived value, and whether the effects met expectations.
- Other Suggestions and Opinions: Elicited open-ended feedback on recommendations for other pet owners, preferred promotion methods, awareness of potential side effects, willingness to pay for higher quality, interest in scientific research data, willingness to participate in educational activities, key purchase motivators, and suggestions for future product development and packaging.

### 2.3 Data analysis

All data were analyzed using JMP Pro statistical software. The analysis consisted of two main parts:

Descriptive Statistics: An analysis was conducted on the entire valid sample (*N* = 328) to summarize demographic characteristics, pet information, and supplement usage experience, providing a comprehensive overview of the sample.

Decision Tree Analysis: To investigate the key predictors influencing the “primary reason” for owners’ supplement choices, a partition analysis model, a form of decision tree, was employed. The model’s outcome variable was the “primary reason for using herbal supplements for pets,” while the predictor variables included “understanding of ingredients and effects,” “perceived benefits for pet health,” and “overall satisfaction.”

**Figure 1:**
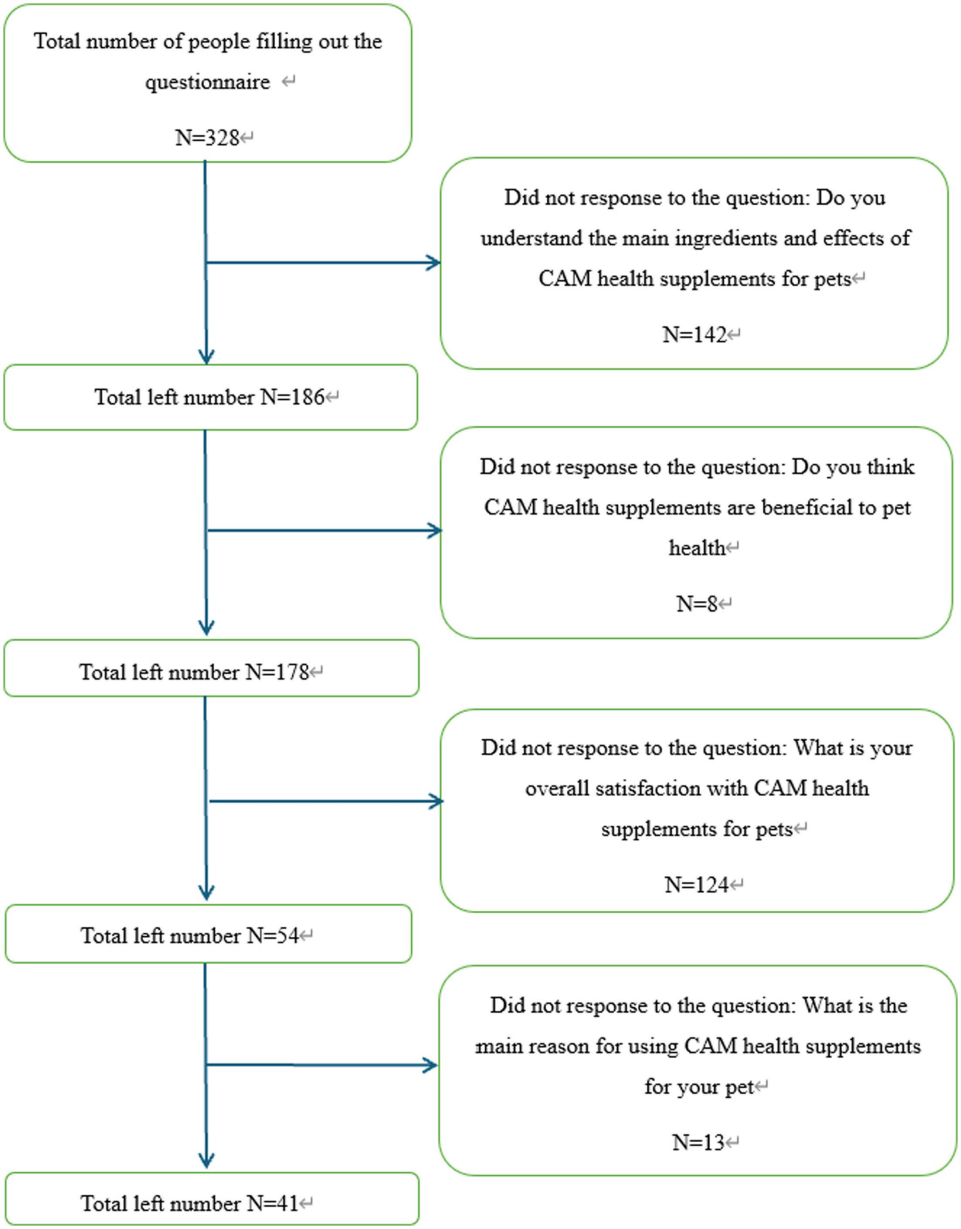
This flowchart details the participant screening process, which began with 328 participants. After sequentially excluding respondents with missing data on key questions, including their understanding of the product (n = 142), perceived benefits (n = 8), overall satisfaction (n = 124), and primary reason for use (n = 13), a final sample of 41 participants was included in the analysis.

To ensure data integrity, the principle of listwise deletion was applied. While this method may reduce the effective sample size, it ensures that only participants with complete and verified cognitive attitude profiles are included in the analysis, thereby maintaining high data fidelity for the predictive model. This screening process is detailed in Figure 1. From the initial 328 samples, we sequentially excluded respondents with missing data on “understanding of ingredients and effects” (*n* = 142), “perceived benefits” (*n* = 8), “overall satisfaction” (*n* = 124), and the outcome variable, “primary reason for use” (*n* = 13). Therefore, the final effective sample size included in the decision tree model analysis was 41 (*n* = 41).

**Figure 2:**
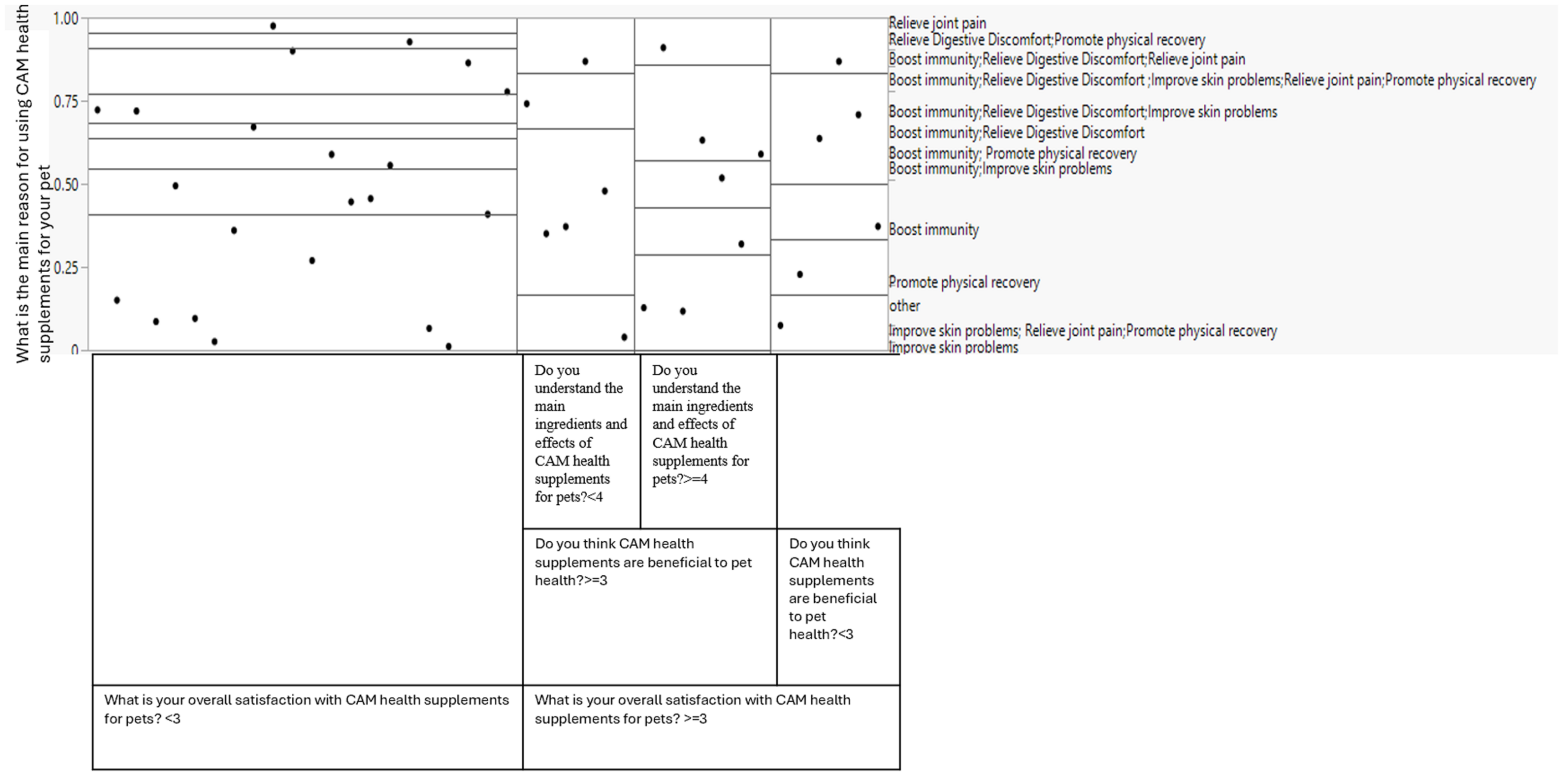
The partition test for classified customers is essential by economic behavior changes. The detailed partition classification was automated and split by the explanation factors and generated the weight of the correlation coefficient.

**Figure 3:**
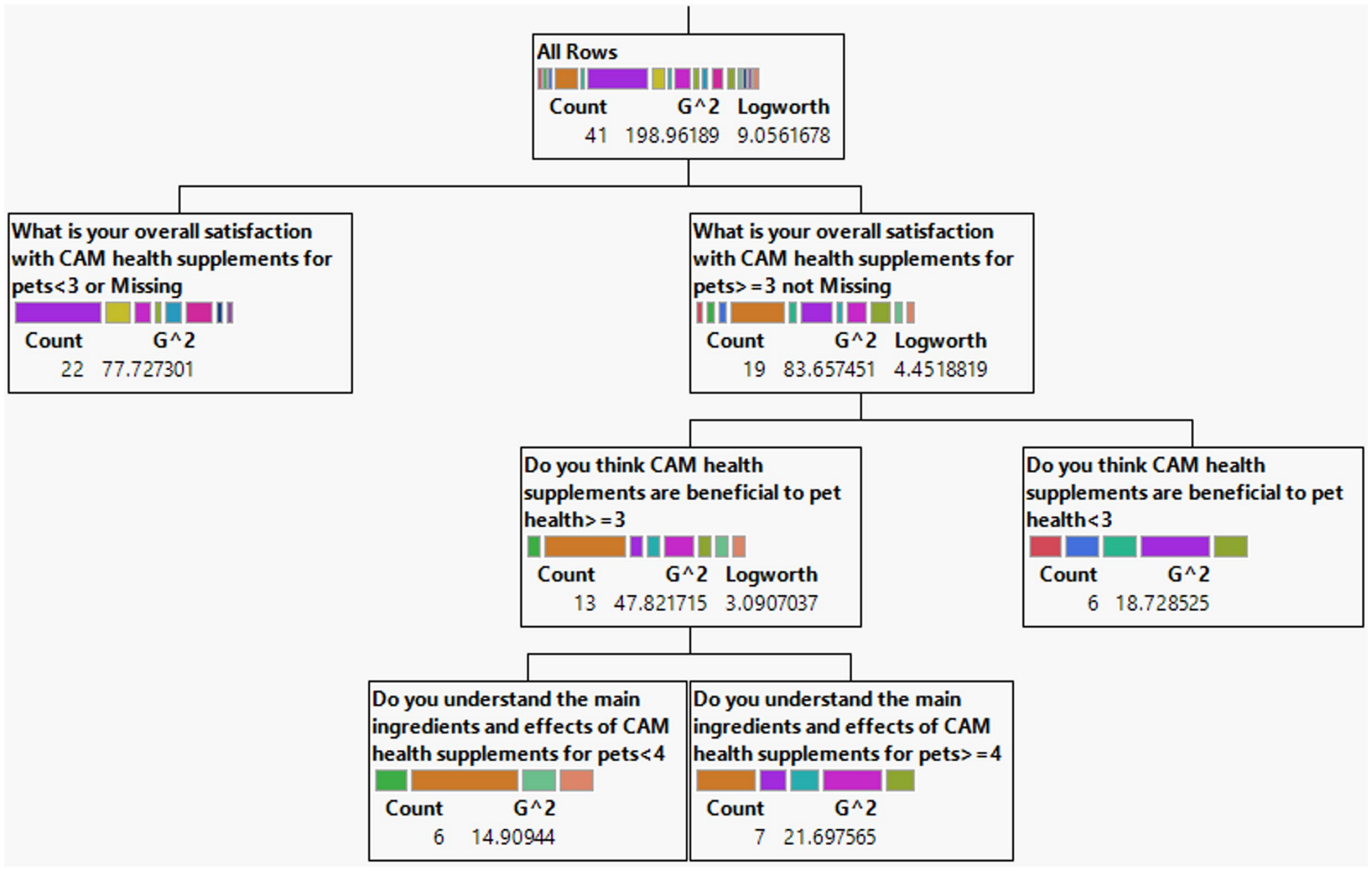
This decision tree analysis identifies ‘overall satisfaction’ as the primary segmenting variable for supplement use. The model further splits users based on ‘perceived benefits’ and ‘understanding of ingredients and effects.’ For instance, among users with high satisfaction (≥3) and a strong belief in benefits (≥3), the level of understanding (≥4 or <4) helps distinguish different user profiles and their motivations, such as using supplements for specific health goals versus general maintenance. Overall, the model reveals a clear hierarchy of cognitive factors that drive user behavior.

**Figure 4:**
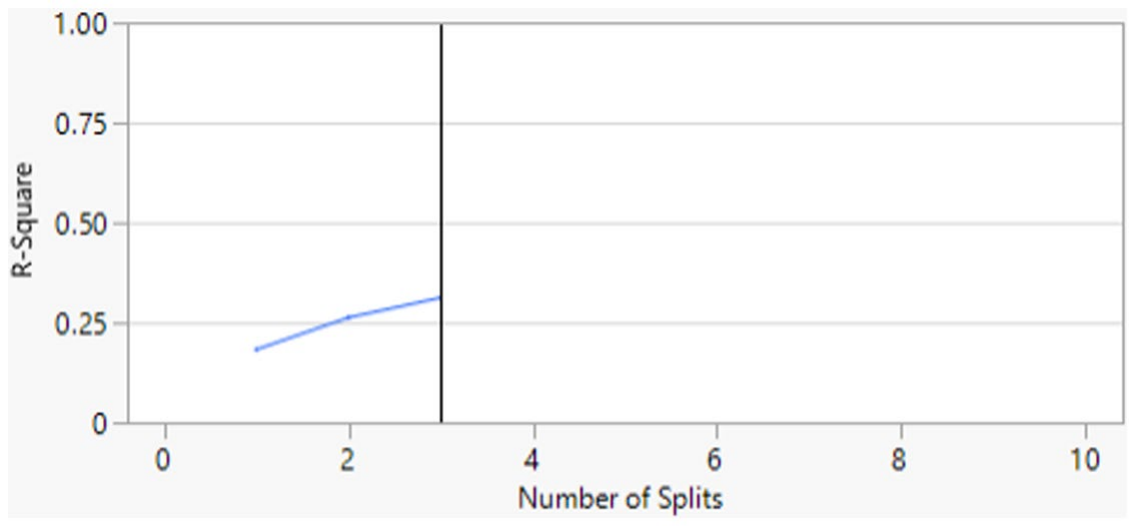
The splitting numbers of the partition test led to the ascending of the correlation coefficient (R-square).

**Figure 5:**
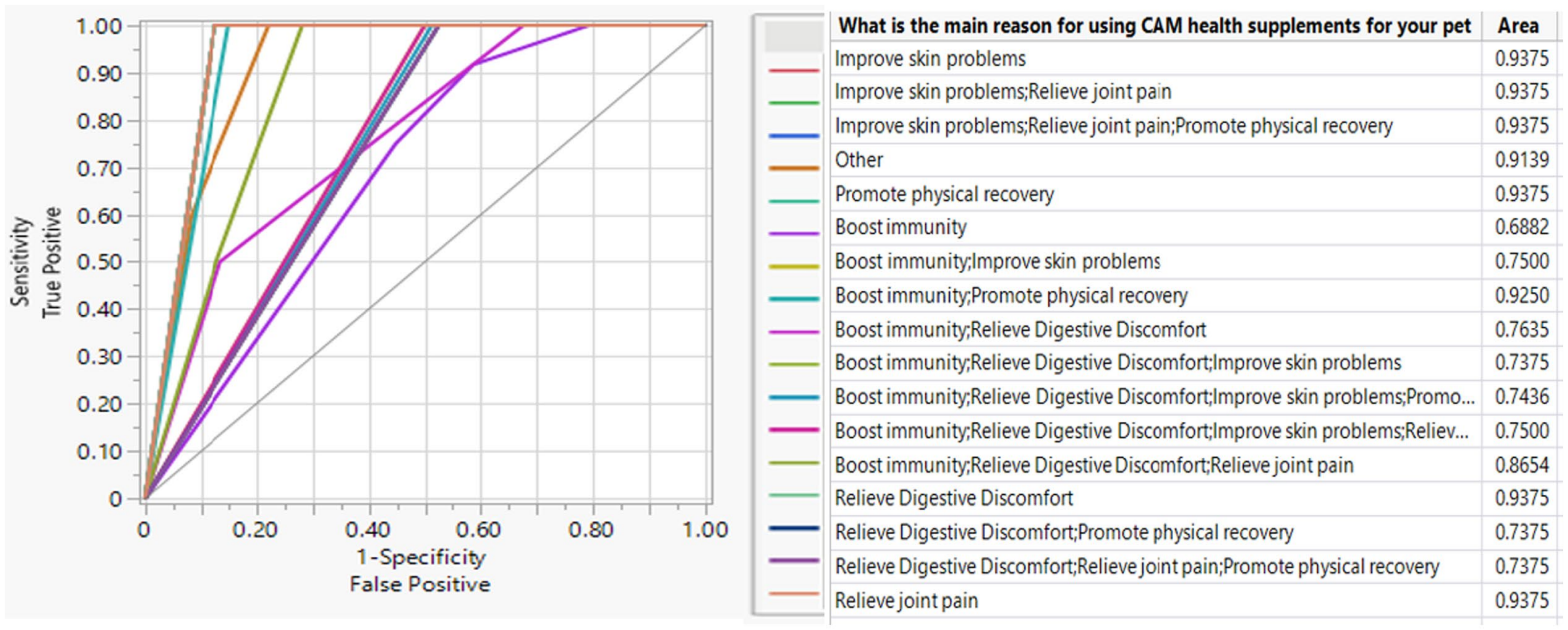
The area under the curve (AUROC) explains three variables affecting the elevation of the AUROC value.

The analysis framework involved four graphical representations to interpret the data: (1) Relationship plots to explore the link between owner cognitive attitudes and reasons for use (Figure 2); (2) A decision tree model to identify key owner segmentation factors (Figure 3); (3) An R-squared plot to evaluate the explanatory power of the decision tree model (Figure 4); and (4) ROC curve plots to assess a predictive model’s ability to discriminate between different reasons for use (Figure 5).

### 2.4 Ethics statement

All components of this study were consistently conducted under the ethical approval of the Ethics Committee of Nihon Pharmaceutical University (No. NPU-EC 6-10). The studies were conducted in accordance with the local legislation and institutional requirements. The participants provided their written informed consent to participate in this study. Specifically, before beginning the questionnaire, all participants were required to read the study’s purpose and instructions and click “I agree to participate in this study” to proceed.

## 3 Results

### 3.1 Part 1: the relationship between owner cognitive attitudes and CAM usage reasons

The relationship plots show a clear association between owners’ cognitive attitude scores and their stated reasons for using CAM supplements (Figure 2). Owners with higher scores (≥3) on “understanding,” “perceived benefits,” and “overall satisfaction” reported reasons for use that were more concentrated on specific health issues, such as enhancing immunity, relieving joint pain, or improving digestive function. In contrast, the group of owners with lower scores (<3) on these dimensions showed a more dispersed, diversified pattern of usage reasons, including general wellness alongside specific issues.

### 3.2 Part 2: key segmentation factors identified via decision tree

The decision tree model identified a hierarchical structure of variables that segment the owner population (Figure 3). The primary segmentation variable was “overall satisfaction,” which split the sample into two main branches: “Satisfaction <3 or Missing” (*N* = 22) and “Satisfaction ≥3” (*N* = 19). Within the high-satisfaction group, the secondary segmentation variable was “perceived benefits,” which further subdivided these owners into “believe in benefits ≥3” (*N* = 13) and “<3” (*N* = 6). The tertiary split occurred among owners who were both highly satisfied and strong believers in the benefits; this split was based on their “level of understanding of ingredients and effects” (using a threshold of 4), separating them into “Understanding <4” (*N* = 6) and “Understanding ≥4” (*N* = 7). This process resulted in at least four distinct terminal owner groups based on these cognitive attitudes.

### 3.3 Part 3: explanatory power of the decision tree model

The R-squared plot illustrates the model’s explanatory power relative to its complexity (Figure 4). The plot shows that as the number of splits increases from 1 to 3, the model’s R-squared value progressively increases from approximately 0.18–0.30. A vertical line at *X* = 3 indicates that the 3-split model was selected for the final analysis. This selected model, driven by satisfaction, perceived benefits, and understanding, explains approximately 30% of the variance in the target variable.

### 3.4 Part 4: predictive performance for user motivations

The set of Receiver Operating Characteristic (ROC) curves evaluated the classification model’s performance in predicting the primary reason for using CAM supplements (Figure 5). The Area Under the Curve (AUC) values were high for several specific reasons: “Relieving joint pain” (AUC = 0.9375), “Relieving digestive discomfort” (AUC = 0.9375), “Improving skin problems” (AUC = 0.9375), and “Promoting physical recovery” (AUC = 0.9375). The model’s predictive performance was lowest when “Boosting immunity” was the sole reason, with an AUC of approximately 0.6882.

## 4 Discussion

There is a growing interest in Complementary and Alternative Veterinary Medicine (CAVM) (Lana et al., 2006). However, uncertainty remains regarding the effectiveness of these therapies. Most treatments are provided by therapists lacking a veterinary background, with only a small fraction administered by animal health professionals, and in some cases, no professionals are involved at all (Bergh et al., 2021). In contrast, Integrative Veterinary Medicine (IVM) has the potential to enhance the health and resilience of animals with acute and chronic diseases through empirically validated and scientifically based treatment strategies (Robinson, 2022). This study aims to analyze pet owners’ perceptions and attitudes, explore their motivations for using Complementary and Alternative Medicine (CAM) products, and identify the key hierarchical drivers influencing usage behavior through decision tree analysis, thereby providing theoretical and practical implications.

### 4.1 Exploration of owner cognition and behavior

The results of this study offer an exploratory perspective on how owners’ intrinsic beliefs shape their purchasing behavior. The study found that owners with positive cognitive attitudes (high satisfaction, high perceived benefits, high understanding) have motivations focused on specific health goals. This suggests that as owners gain more information and see positive effects, they shift towards a more “goal-oriented” usage model. The terminology in this study is standardized to refer to CAM for specific products and CAVM for the broader veterinary field. Research has indicated that owners’ “perceived behavioral control” and “attitudes” toward CAVM can predict their actual use, and veterinarians play a key role in providing objective, accurate information about CAVM (Keller et al., 2023). This aligns with the findings of this study, which show a significant positive correlation between owners’ positive cognitive attitudes and their specific motivations for using CAM products.

Furthermore, past research has shown that 76% of owners have used some form of therapy, with nutritional supplements being the most common (Lana et al., 2006). This is consistent with the present study, where respondents tended to use CAM products as daily supplements, emphasizing “holistic health” rather than specific therapeutic effects (Figure 2). Human behavior is often influenced by perceptual and cognitive assessments of the object of action; categorization processes highlight discernible features while ignoring irrelevant factors (Frank et al., 2023). The decision tree model aptly reflects this cognitive blueprint by establishing a hierarchical structure (Song and Lu, 2015).

The decision tree analysis (Figure 3) further elucidates the hierarchical relationships between these cognitions. The model identifies “overall satisfaction” as the primary splitting variable, a key finding indicating it is the most powerful indicator for distinguishing among different owner groups. This implies that satisfaction is a “threshold cognition”: only after a positive experience of satisfaction is established do other attitudes (such as belief in efficacy or understanding of ingredients) further influence usage behavior. This hierarchical structure supports existing health behavior theories (e.g., TPB, HBM), illustrating that behavior is not driven by a single belief but by the result of multiple interacting cognitive chains, with satisfaction playing a foundational role (Ratz and Lippke, 2022). As owners accumulate knowledge and perceive positive effects, their behavior tends to shift from exploratory to confident, problem-solving-oriented use. Conversely, owners with lower cognitive scores exhibit dispersed motivations, possibly remaining in a “trial phase,” experimenting with products to maintain general health but not yet convinced of their efficacy for specific conditions. Their low satisfaction may stem from results that do not meet expectations, preventing them from identifying the primary reason for using the product.

### 4.2 Significance of the predictive model and industrial application value

To delve deeper into which owner characteristics or attitudes best differentiate CAM users, this study constructed and analyzed a decision tree model. Since its introduction in the 1960s, the decision tree has been one of the most effective tools in data mining (Hastie et al., 2009). It partitions samples into a hierarchical structure based on multiple potential influencing factors through binary splits (Song and Lu, 2015) and screens for the most predictive variables. The results show that owner satisfaction, perceived benefits, and understanding do not influence behavior independently; rather, they shape the CAM usage profile in a conditionally dependent manner (Segal, 1988).

The model’s performance, assessed via ROC curves (Figures 4, 5), reveals which owner motivations are most distinctive. The model demonstrates extremely high predictive accuracy for joint, digestive, and skin issues (AUC > 0.93), indicating these are clear market needs. Examples include common canine conditions like atopic dermatitis (Beynen et al., 2011), osteoarthritis (Anderson et al., 2018), and inflammatory bowel disease (Cave et al., 2023). This group likely represents the core users of the CAM market, exhibiting more rational, problem-solving-oriented behavior. In contrast, the low predictability of “enhancing immunity” is noteworthy. Although there is abundant evidence in nutrition research on immunity (Herrera et al., 2019; Williams et al., 2020; Rodrigues et al., 2022), its predictive AUC was relatively low. This suggests that the profile of owners motivated solely by this factor is not unique and exhibits higher heterogeneity. This may imply that “enhancing immunity” is a broader, more preventative goal adopted by a wider variety of users. Therefore, its market positioning in companion animals still needs to be reinforced through clearer marketing messages (Di Cerbo et al., 2017; Baritugo et al., 2023).

Beyond its explanatory power, this model also holds significant practical value for the pet supplement industry, particularly in market segmentation and marketing strategy (Wang, 2025):

1. Data-Driven Customer Segmentation: This study provides a market segmentation framework centered on psychological characteristics, capable of distinguishing groups such as “Convinced Users” (high satisfaction, high belief, high understanding) and “Cautious New Users” (low satisfaction, dispersed motivation).
2. Customized Marketing and Communication: The needs of different segments vary significantly. “Convinced Users” prefer scientific data and loyalty programs, whereas “Cautious New Users” require educational content, testimonials, and introductory offers to build trust.
3. Product Development and Positioning: The model’s high predictive accuracy for joint, digestive, and skin problems shows these are clear market needs.

Overall, this study emphasizes the central role of the owner’s “subjective cognition” in CAM usage behavior. As demand increases, veterinarians and related professionals should be prepared to respond to diverse questions about CAVM (Bergh et al., 2021) and provide neutral, scientific information. These results also echo health behavior theories such as the TPB and HBM (Resnicow et al., 1999; Song and Lu, 2015), confirming that CAM use is not driven by a single motive but is the result of an interaction of multi-level cognitive factors.

### 4.3 Limitations and future research

This study is exploratory and has several limitations. First, the sample size is relatively small (*N* ≈ 40), and participants were limited to students from a single medical university in Taiwan. Additionally, the cross-sectional design and self-report questionnaires may introduce recall bias and limit causal inferences and the generalizability of the results. However, these limitations pave the way for future research. Future studies could be considered:

- Longitudinal Studies: Tracking new users over time to observe how satisfaction, beliefs, and understanding evolve.
- Qualitative Exploration: Using in-depth interviews to deeply understand the nuanced motivations of the “enhancing immunity” group.
- Expanded Sample Validation: Repeating the study with more diverse populations.
- Interventional Studies: Conducting A/B testing to design tailored marketing campaigns.

This study is represented as primarily exploratory. The decision tree model, while showing high predictive performance for joint and digestive issues (AUC = 0.9375), is based on a refined sample of 41 participants from a specific student demographic at a single medical university. Therefore, the results may not be generalizable to the broader population. The lower AUC for “boosting immunity” (approx. 0.69) suggests high behavioral heterogeneity in this area, necessitating further qualitative or longitudinal research to refine user profiles. Additionally, according to Wang (2025), future consumer behavior analysis could benefit from integrated decision tree and association rule models for larger datasets.

## 5 Conclusion

This study systematically investigated motivations, cognitive attitudes, and interactions among pet owners who use CAM supplements. The main conclusions include:

1. Owners’ overall satisfaction, perceived benefits, and understanding of the products are key factors influencing CAM use and differentiating user groups.
2. Overall satisfaction plays a primary role in market segmentation and is a prerequisite for forming more targeted usage patterns.
3. Building on a foundation of high satisfaction, perceived benefits, and greater understanding further distinguishes owners, forming unique user profiles.
4. Owners with high cognition (high satisfaction, high belief, high understanding) tend to use CAM for specific health problems (such as joint, digestive, and skin diseases), and the predictive model identifies these with high accuracy.
5. The model’s ability to predict “enhancing immunity” as a singular motivation is relatively weak, indicating that the user profile associated with this goal is more diverse.

These findings have significant practical implications. To increase owner acceptance of CAM products, differentiated strategies should be designed based on cognitive structures, such as strengthening the dissemination of scientific knowledge to promote understanding or enhancing perceived benefits through clinical case sharing. At the marketing level, products can be prioritized for promotion to the “clear health need” group, while providing education and support to the “prevention-oriented” group.

## Data Availability

The original contributions presented in the study are included in the article/supplementary material, further inquiries can be directed to the corresponding author.
