# Supplementary material for: Machine learning analysis of student consumer choices for pet supplements
Source: Front Artif Intell. 2026 Jun 3;9:1764233. doi: 10.3389/frai.2026.1764233 (PMC13272473; doi:10.3389/frai.2026.1764233)
Supplement: Supplementary file 1 [file Supplementary_File_1.docx]

Supplementary Material

Supplemental Table 1. Pet Owner Survey on the Use of Complementary and Alternative Medicine (CAM) Health Supplements for Pets

**I. SURVEY DESCRIPTION**

Dear Respondent,
This questionnaire aims to understand pet owners' perceptions and usage of Complementary and Alternative Medicine (CAM) health supplements for their pets.
Your responses are crucial to our research. This survey is completely anonymous, and all data will be used for academic research purposes only.
It will take approximately 15-20 minutes to complete. Thank you for your participation!

Pets’ owner: Yes: [ ] No: [ ]

**II. BASIC INFORMATION OF OWNER**

**1. Age?**

1. Under 18 years old 2. 18-25 years old 3. 26-35 years old 4. 36-45 years old 5. 46-55 years old 6. Over 56 years old

**2. Gender?**

1. Male 2. Female 3. Other (Please specify:__________)

**3. Education level?**

1. High school diploma or below 2. Bachelor's degree 3. Master's degree 4. Doctoral degree

**4. You and your pet's location of residence?**

1. County/City: _________ 2. Town/District: _________

**5. Occupation?**

1. Student 2. Office worker/White-collar 3. Self-employed/Entrepreneur 4. Civil servant 5. Retired 6. Other (Please specify: _________)

**6. Annual income?**

1. Under NT$400,000 2. NT$410,000 - NT$700,000 3. NT$710,000 - NT$1,000,000 4. NT$1,010,000 - NT$1,300,000 5. Above NT$1,310,000

**III. PET INFORMATION OF OWNER**

**7. Your total years of pet ownership experience?**

1. Less than 1 year 2. 1-3 years 3. 4-6 years 4. 7-10 years 5. Over 10 years

**8. Do you currently have any pets?**

1. Yes 2. No

**9. How many pets do you currently own in total? (Number of cats and dogs)**

1. 1 2. 2 3. 3 4. 4 5. 5 or more

**10. If you have pets, how many cats do you own?**

1. 0 2. 1 3. 2 4. 3 5. 4 or more

**11. What breed(s) are your cat(s)? (Multiple choices possible)**

1. Mixed breed 2. Maine Coon 3. Ragdoll 4. British Shorthair 5. American Shorthair 6. Persian 7. Munchkin 8. Norwegian Forest Cat 9. Scottish Fold 10. Other (Please specify:_______)

**12. What is the current health status of your cat(s)? (Multiple choices possible)**

1. Very healthy 2. Skin and coat problems 3. Urinary tract problems 4. Kidney problems 5. Digestive problems 6. Emotional problems 7. Joint problems 8. Heart problems 9. Eye problems 10. Other (Please specify:________)

**13. If you have pets, how many dogs do you own?**

1. 0 2. 1 3. 2 4. 3 5. 4 or more

**14. What size(s) are your dog(s)? (Multiple choices possible)**

1. Miniature (under 4kg) 2. Small (5-10kg) 3. Medium (11-25kg) 4. Large (over 25kg)

**15. What breed(s) are your dog(s)? (Multiple choices possible)**

1. Chihuahua 2. Pomeranian 3. Yorkshire Terrier 4. Maltese 5. Poodle 6. French Bulldog 7. Dachshund 8. Shiba Inu 9. Beagle 10. Labrador Retriever 11. Golden Retriever 12. Siberian Husky 13. Other (Please specify:___________)

**16. What is the current health status of your dog(s)? (Multiple choices possible)**

1. Very healthy 2. Skin and coat problems 3. Urinary tract problems 4. Kidney problems 5. Digestive problems 6. Emotional problems 7. Joint problems 8. Heart problems 9. Eye problems 10. Other (Please specify:________)

**IV. AWARENESS AND ACCEPTANCE OF CAM HEALTH SUPPLEMENTS FOR PETS**

**17. Have you heard of CAM health supplements for pets?**

1. Yes 2. No

**18. How did you learn about CAM health supplements for pets? (Multiple choices possible)**

1. Introduced by friends and family 2. Recommended by a veterinarian 3. Online information 4. Social media 5. Pet store 6. Other (Please specify:________)

**19. Do you think CAM health supplements are beneficial to pet health?**

1. Very helpful 2. Helpful 3. Neutral 4. Not helpful 5. Not helpful at all

**20. Do you understand the main ingredients and effects of CAM health supplements for pets?**

1. Very well 2. Somewhat 3. Neutral 4. Not really 5. Not at all

**21. Are you willing to use CAM health supplements for your pet's health?**

1. Not at all willing 2. Not very willing 3. No preference 4. Relatively willing 5. Very willing

**V. ACTUAL USAGE OF CAM HEALTH SUPPLEMENTS FOR PETS**

**22. Have you ever used CAM health supplements for your pet's health?**

1. Never used 2. Occasionally used 3. Sometimes used 4. Often used 5. Always used

**23. Name(s) of CAM health supplement product(s) you have used:**

_________

**24. Are you currently using CAM health supplements for your pet?**

1. Yes 2. No

**25. If yes, how long have you been using them?**

1. Less than 1 month 2. 1-3 months 3. 4-6 months 4. 7-12 months 5. Over 1 year

**26. What is the frequency of your use of CAM health supplements?**

1. Never use 2. Occasionally (less than once a month) 3. Sometimes (1-2 times a month) 4. Often (1-2 times a week) 5. Frequently (daily)

**27. Where do you usually purchase CAM health supplements for your pet?**

1. Veterinary clinic 2. Online shopping 3. Pet store 4. Other (Please specify: _____)

**28. What is the main reason for using CAM health supplements for your pet? (Multiple choices possible)**

1. Boost immunity 2. Relieve Digestive Discomfort 3. Improve skin problems 4. Relieve joint pain 5. Promote physical recovery 6. Other (Please specify: __________)

**29. What is your approximate monthly expenditure on CAM health supplements for your pet?**

1. Less than NT$100 2. NT$100-300 3. NT$301-500 4. NT$501-1000 5. Over NT$1000

**VI. EVALUATION OF CAM HEALTH SUPPLEMENT EFFECTS**

**30. After using CAM health supplements, do you think your pet's health has improved?**

1. No improvement at all 2. Very little Improvement 3. Slight improvement 4. Obvious improvement 5. Significant improvement

**31. Do you think CAM health supplements help to boost your pet's immunity?**

1. No improvement at all 2. Very little Improvement 3. Slight improvement 4. Obvious improvement 5. Significant improvement

**32. Do you think CAM health supplements have improved your pet’s digestive function?**

1. No improvement at all 2. Very little Improvement 3. Slight improvement 4. Obvious improvement 5. Significant improvement

**33. Do you think CAM health supplements have improved your pet's skin and coat condition?**

1. No improvement at all 2. Very little improvement 3. Slight improvement 4. Obvious improvement 5. Significant improvement

**34. Do you think CAM health supplements have helped relieve your pet's stress?**

1. No improvement at all 2. Very little Improvement 3. Slight improvement 4. Obvious improvement 5. Significant improvement

**35. What dosage form of CAM health supplements do you find most convenient for feeding your pet?**

1. Powder 2. Capsule 3. Concentrated liquid 4. Tablet 5. Other (Please specify:____________)

**36. What is your overall satisfaction with CAM health supplements for pets?**

1. Very satisfied 2. Satisfied 3. Neutral 4. Dissatisfied 5. Very dissatisfied

**37. How do you feel about the price of CAM health supplements for pets?**

1. Very expensive 2. Expensive 3. Reasonable 4. Cheap 5. Very cheap

**38. What price range are you willing to accept for CAM health supplements for pets?**

1. Under NT$300 2. NT$300-800 3. NT$800-1300 4. NT$1300-1800 5. Above NT$1800

**39. Do you think the effects of CAM health supplements meet your expectations?**

1. Completely meet 2. Meet 3. Neutral 4. Do not meet 5. Do not meet at all

**VII. OTHER SUGGESTIONS AND OPINIONS**

**40. Would you recommend CAM health supplements to other pet owners?**

1. Yes 2. Maybe 3. Not sure 4. Probably not 5. No

**41. What do you think is the best way to promote CAM health supplements for pets? (Multiple choices possible)**

1. Online Advertising 2. Social media 3. Pet exhibitions 4. Veterinarian recommendations 5. Other

**42. Are you aware of the potential side effects of CAM health supplements for pets?**

1. Very aware 2. Aware 3. Neutral 4. Not aware 5. Not at all aware

**43. Are you willing to pay a higher price for higher quality CAM health supplements for pets?**

1. Very willing 2. Willing 3. Neutral 4. Unwilling 5. Not at all willing

**44. Do you want to have access to more scientific research data on CAM health supplements for pets?**

1. Very much 2. Somewhat 3. Neutral 4. Not really 5. Not at all

**45. Have you ever attended a lecture or seminar related to CAM health supplements for pets?**

1. Yes 2. No

**46. If given the opportunity, would you be willing to participate in such activities?**

1. Very willing 2. Willing 3. Neutral 4. Unwilling 5. Not at all willing

**47. What is the main reason that attracts you to purchase CAM health supplements?**

1. Efficacy 2. Price 3. Safety 4. All-natural 5. Other (Please specify:____________)

**48. What suggestions or opinions do you have about CAM health supplements for pets?**

Please specify: ___________________

**49. What types of CAM health supplements for pets do you think should be developed in the future?**

Please specify:_____________________

**50. What suggestions do you have for the packaging of CAM health supplements for pets?**

Please specify:_______________________

**SURVEY CONCLUSION**

Thank you very much for taking the time to complete this questionnaire! Your feedback is valuable to our research.
If you have any questions or would like to learn more, please feel free to contact us.
We wish you and your pet good health and happiness!
